# Predicted Strain Coverage of a New Meningococcal Multicomponent Vaccine (4CMenB) in Spain: Analysis of the Differences with Other European Countries

**DOI:** 10.1371/journal.pone.0150721

**Published:** 2016-03-07

**Authors:** Raquel Abad, Verónica Medina, Maria Stella, Giuseppe Boccadifuoco, Maurizio Comanducci, Stefania Bambini, Alessandro Muzzi, Julio A. Vázquez

**Affiliations:** 1 Reference Laboratory for Meningococci, National Centre for Microbiology, Instituto de Salud Carlos III, Majadahonda, Madrid, Spain; 2 Novartis Vaccines and diagnostics, a GSK Company, Siena, Italy; University of Padova, Medical School, ITALY

## Abstract

**Background:**

A novel meningococcal multicomponent vaccine, 4CMenB (Bexsero^®^), has been approved in Europe, Canada, Australia and US. The potential impact of 4CMenB on strain coverage is being estimated by using Meningococcal Antigen Typing System (MATS), an ELISA assay which measures vaccine antigen expression and diversity in each strain. Here we show the genetic characterization and the 4CMenB potential coverage of Spanish invasive strains (collected during one epidemiological year) compared to other European countries and discuss the potential reasons for the lower estimate of coverage in Spain.

**Material and Methods:**

A panel of 300 strains, a representative sample of all serogroup B *Neisseria meningitidis* notified cases in Spain from 2009 to 2010, was characterized by multilocus sequence typing (MLST) and FetA variable region determination. 4CMenB vaccine antigens, PorA, factor H binding protein (fHbp), *Neisseria* Heparin Binding Antigen (NHBA) and Neisserial adhesin A (NadA) were molecularly typed by sequencing. PorA coverage was assigned to strain with VR2 = 4. The levels of expression and cross-reactivity of fHbp, NHBA and NadA were analyzed using MATS ELISA.

**Findings:**

Global estimated strain coverage by MATS was 68.67% (95% CI: 47.77–84.59%), with 51.33%, 15.33% and 2% of strains covered by one, two and three vaccine antigens, respectively. The predicted strain coverage by individual antigens was: 42% NHBA, 36.33% fHbp, 8.33% PorA and 1.33% NadA. Coverage within the most prevalent clonal complexes (cc) was 70.37% for cc 269, 30.19% for cc 213 and 95.83% for cc 32.

**Conclusions:**

Clonal complexes (cc) distribution accounts for variations in strain coverage, so that country-by-country investigations of strain coverage and cc prevalence are important. Because the cc distribution could also vary over time, which in turn could lead to changes in strain coverage, continuous detailed surveillance and monitoring of vaccine antigens expression is needed in those countries where the multicomponent vaccine is introduced. This is really important in countries like Spain where most of the strains are predicted to be covered by only one vaccine antigen and the chance for escape mutants to emerge with vaccine use is higher. Based on the observed data, cc213 should receive special attention as it is associated with low predicted strain coverage, and has recently emerged in Spain.

## Introduction

*Neisseria meningitidis* is the most common cause of bacterial meningitis and a major cause of septicaemia worldwide [1–3]. Although there are 12 *N*. *meningitidis* serogroups currently described, defined on the basis of capsular polysaccharide type, only six (A, B, C, W, X, Y) cause life-threatening disease [4].

Serogroup B is the most frequently observed serogroup in industrialized countries, causing a third of the cases in the United States [5] and more than 70% in Europe [6]. In the epidemiological year 2012–2013, a total of 271 confirmed cases of invasive meningococcal disease (IMD), either by isolation or by real time PCR, were reported in Spain with an incidence rate of 0.59 per 100.000 population, with serogroup B being responsible for 71.2% IMD [7].

A major advance in meningococcal disease prevention has been the development of meningococcal conjugate vaccines, including meningococcal serogroup C glycoconjugate vaccines and quadrivalent conjugate vaccines against serogroups A, C, Y and W [8]. Unlike serogroups A, C, Y and W, serogroup B has presented unique challenges to vaccine development. A major obstacle to the development of a serogroup B meningococci (MenB) vaccine is that the polysaccharide capsule mimics the human neural cell adhesion molecule [9] so the use of other antigens has been necessary.

Different subcapsular targets have been investigated since the 1980s, either as outer membrane vesicle (OMV) or as individual antigens [10]. Several candidate OMV vaccines have been developed and tested in large-scale efficacy studies in Norway [11], Cuba [12], Brazil [13], Chile [14, 15], New Zealand [16], and Normandy (France) [17]. These OMV vaccines have proven effective in the control of serogroup B outbreaks, however the serum bactericidal antibody response induced is largely specific to the serosubtype, and do not provide protection against strains carrying PorA that differ from that in the vaccine [17,18]. The need to find a universal MenB vaccine has led to alternative strategies like reverse vaccinology which utilizes genomic information to identify new vaccine candidates.

Reverse vaccinology has enabled the identification of several non-capsular protein surface antigens: Neisserial adhesin A (NadA), *Neisseria* Heparin Binding Antigen (NHBA) and factor H binding protein (fHbp) [19]. These three antigens, along with the OMV-based vaccine MeNZB (from *N*. *meningitidis* strain NZ98/254, expressing PorA serosubtype P1.4), have been combined into a multicomponent vaccine against MenB, 4CMenB (Bexsero^®^). This new vaccine has been approved for use in people 2 months of age and above by the European Commission in January 2013, it has also been approved in Australia and Canada, and it was recently approved for use in individuals from 10 to 25 years of age by the FDA in January 2015 [20]. To determine the potential impact of this novel multicomponent protein-based vaccine on endemic MenB bacteria in different countries or regions, a new method (Meningococcal Antigen Typing System MATS) has been developed. MATS combines conventional genotyping for PorA with a specialized sandwich enzyme-linked immunosorbent assay (ELISA) that determines phenotypic expression and the cross-reactivity, or relative potency (RP), of fHbp, NadA, and NHBA on individual strains [21].

MATS has been initially used to assess vaccine coverage on invasive MenB strains isolated mostly during one epidemiological year (2007/2008) in five European countries, England and Wales, France, Germany, Italy, and Norway [22]. More recently, MATS has been applied to many other strain collections thus providing estimates potential vaccine coverage in many countries worldwide. Here we show the genetic characterization and the estimated coverage of strains isolated in Spain in comparison with the previously tested European countries. We also present a potential explanation of the lower value for Spain by providing a detailed breakdown of the MATS results and the genetic properties of 300 invasive MenB strains isolated in Spain from 2009 to 2010.

## Material and Methods

### Bacterial Strains

A total of 300 invasive MenB isolates received at the Spanish National Reference Laboratory (NRL) were included in this study. The isolates were recovered from blood or cerebrospinal fluid of patients with meningococcal disease in Spain. The NRL performs functions to support the National Health System and as such had access, through a sample management system, to identifying patient information. The strains were collected for this study as follows:

- All invasive MenB isolates received at the Spanish NRL from January 2009 to June 2009 (150 strains).
- From July 2009 to June 2010 the isolates were collected systematically, with every third invasive MenB strains collected by the Spanish NRL (150 strains).

The meningococcal isolates received at the Spanish NRL comprise 80–85% of all notified invasive meningococcal disease cases within Spain. Thus, the isolates included in this set are a good representation of all MenB notified cases in Spain from January 2009 to June 2010, the period covered by the study, and included isolates from all the Spanish Autonomous Communities.

All *N*. *meningitidis* isolates received in the Spanish NRL are routinely serogrouped by slide agglutination with specific polyclonal antibodies.

### Genetic Characterization (MLST, PorA, FetA)

Multilocus sequence typing (MLST) was performed as described by Maiden *et al*. [23], on all 300 MenB *N*. *meningitidis* isolates included in the study. For *N*. *meningitidis*, PCR-amplified DNAs from 7 different housekeeping genes (*abcZ*, *adk*, *aroE*, *fumC*, *gdh*, *pdhC*, and *pgm*) were sequenced and compared with the Neisseria MLST database (http://pubmlst.org/neisseria/). Sequence Type (ST) and clonal complex (cc) were assigned according to the same web site.

PorA genotyping was done by sequencing the variable regions (VRs) of *porA* gene according to methods described previously [24] and compared with accessions in the Neisseria PorA database (http://pubmlst.org/neisseria/PorA/).

The FetA variable region was also obtained for all studied strains as described in Thompson *et al*. [25]. Those sequences were compared with accessions in the Neisseria FetA database (http://pubmlst.org/neisseria/FetA/).

### 4CMenB/Bexsero^®^ vaccine antigens molecular typing

4CMenB/Bexsero^®^ vaccine antigens genes (*fHbp*, *nhba* and *nadA*) were PCR amplified and sequenced as previously described [26]. Alleles and the corresponding protein variants were assigned using the PubMLST Neisseria sequence typing database (http://pubmlst.org/neisseria/).

### Meningococcal antigen typing system (MATS)

The levels of expression and cross-reactivity of fHbp, NadA, and NHBA were analyzed in all MenB isolates using the MATS ELISA according to the method previously described [21]. For each isolate the MATS reactivity or relative potency (RP) of each of three antigens was obtained by mathematically comparing the serial dilution curve of each *N*. *meningitidis* isolate to that obtained using a reference MenB strain. In a previous study [21] a minimum level of RP, named the positive bactericidal threshold (PBT), for each of the three antigens was determined (fHbp: 0.021, NHBA: 0.294, NadA: 0.009). The PBT is the minimum level of RP required for an isolate to be considered susceptible to killing in the human serum bactericidal antibody assay (hSBA) by antibodies induced by 4CMenB/Bexsero^®^ [21].

### Estimation of MenB strain coverage

Previously [21] has been demonstrated (by correlation with bactericidal effect in the serum bactericidal antibody assay with human complement by pooled sera taken after immunization) than the presence of at least one of the three antigens with MATS RP greater than PBT or the presence of PorA P1.4 is enough to be an isolate covered by the vaccine. Strains that did not meet these criteria were deemed not covered. In this study the predicted strain coverage was defined as the proportion of isolates with RP greater than the PBT for one or more vaccine antigens, with PorA P1.4, or both.

### Statistical analysis

As described in the MATS interlaboratory standardization study [27], empirical estimates of the 95% CIs for the positive bactericidal thresholds were derived with a lognormal approximation based on overall assay reproducibility (0·014–0·031 for fHbp, 0·004–0·019 for NadA, and 0·169–0·511 for NHBA). These values were used to define the 95% CIs of strain coverage.

## Results

### Genetic Characterization (MLST, PorA, FetA)

**Fig 1:**
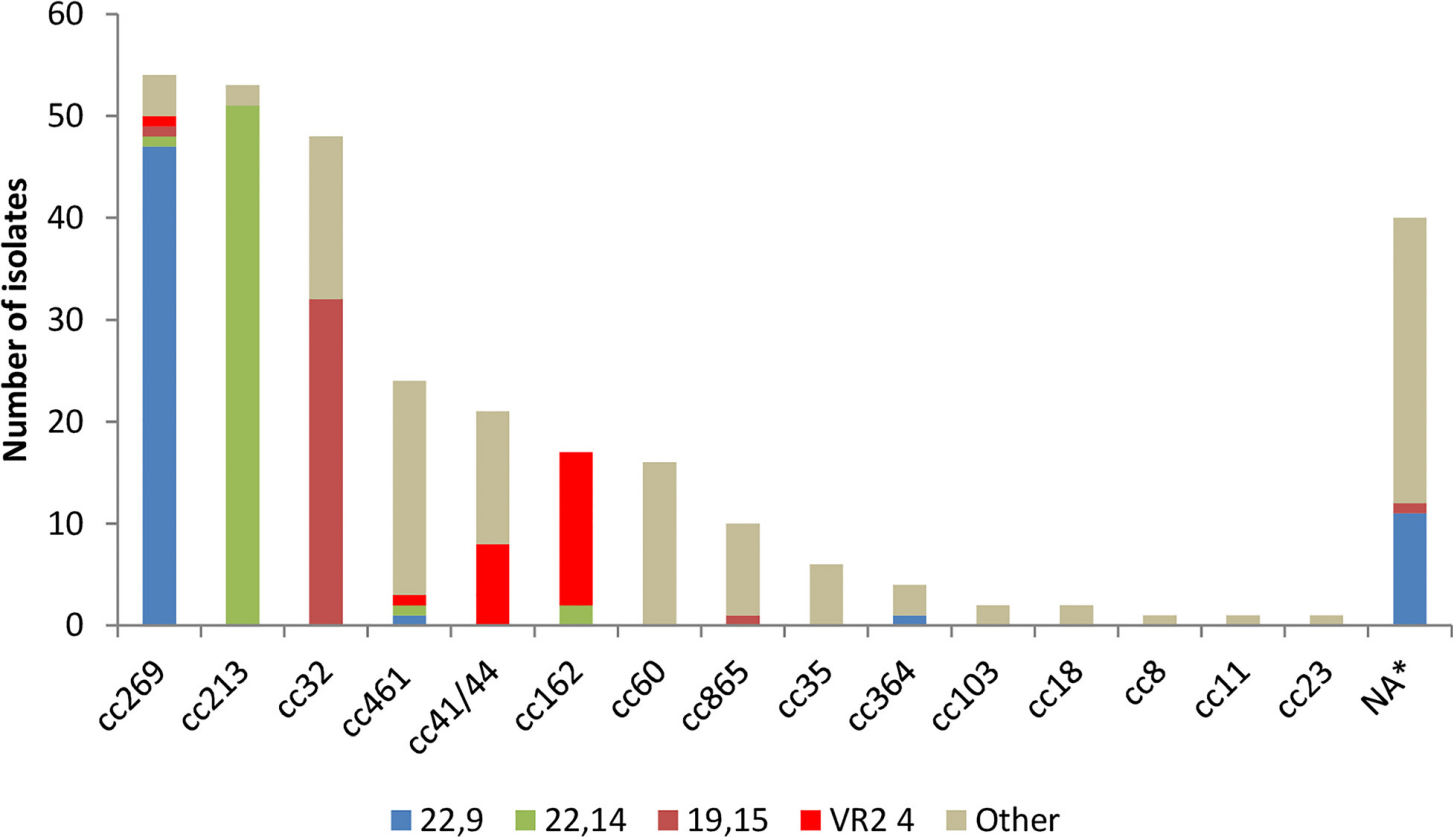
Distribution of clonal complexes and main PorA genotypes in the 300 isolates panel. *NA: Clonal complex non assigned.

Of the 300 studied invasive MenB isolates, 86.66% (n = 260) belonged to 16 different clonal complexes, and the remaining 13.33% (n = 40) did not belong to any assigned clonal complex (NA) (Fig 1). Clonal complexes 269 (n = 54, 18%), 213 (n = 53, 17.67%) and 32 (n = 48, 16%) accounted for most of the strains (n = 155, 51.67%). Although 12 different sequence types (STs) were identified among isolates belonging to the cc269, most of the strains belonged to ST-1163 (n = 34). A similar distribution was observed with the other two major clonal complexes, 213 and 32. In both cases 17 different STs were found: among the cc213 most of the strains belonged to either ST-213 (n = 16) or ST-3496 (n = 18) and, in the cc32 the most frequent ST was ST-749 (n = 19) (S1 Table).

We identified 24 different PorA VR1 variants and 33 different VR2 variants in the 300 strains panel. The most frequent VR1 variants were 22 (n = 119, 39.67%), 19 (n = 43, 14.33%) and 7–2 (n = 38, 12.67%). As for VR2, the most frequent variants were 14 (n = 70, 23.33%), 9 (n = 65, 21.67%) and 15 (n = 39, 13%). The PorA VR2 variant 4, one of the 4CMenB/Bexsero^®^ vaccine components, was present in 25 (8.33%) of the 300 isolates, 15 belonged to the cc162 (PorA genotype 7–2,4), 8 to cc41/44 (PorA genotype 7–2,4), 1 to cc269 (PorA genotype 21,4) and 1 to cc461 (PorA genotype 19–2,4) (Fig 1).

There were 55 different PorA genotypes (PorA VR1,VR2) identified, of which only 25 were present in more than one isolate (S1 Table). The three most frequent PorA genotypes were 22,9 (n = 60, 20%), 22,14 (n = 55, 18.33%) and 19,15 (n = 35, 11.67%). PorA genotype 22,9 was predominant in the cc269, genotype 22,14 in the cc213 and 19,15 in the cc32 (Fig 1).

The FetA variable region (VR) was sequenced in 298 isolates (S1 Table), no *fetA* amplicon was obtained in two cases. Although 29 different FetA VR were observed, 12 were only present once and 3 twice. F5-5 was the most frequently FetA VR observed (n = 49, 16.33%) and was predominant among genotype 22,14 cc213 strains. F5-1 and F1-55 were present in 32 (10.67%) and 30 (10%) isolates, respectively. F5-1 was associated with genotype 19,15 cc32 strains, and F1-55 with genotype 22,9 cc269 strains.

### 4CMenB/Bexsero^®^ vaccine antigens molecular typing

The *fHbp* gene was present in all isolates. FHbp variant family 1, 2 and 3 peptides were found; in detail, a total of 72 different fHbp peptides were identified (37 variant family 1, 12 variant family 2, and 23 variant family 3 peptides) (S1 Table).

**Fig 2:**
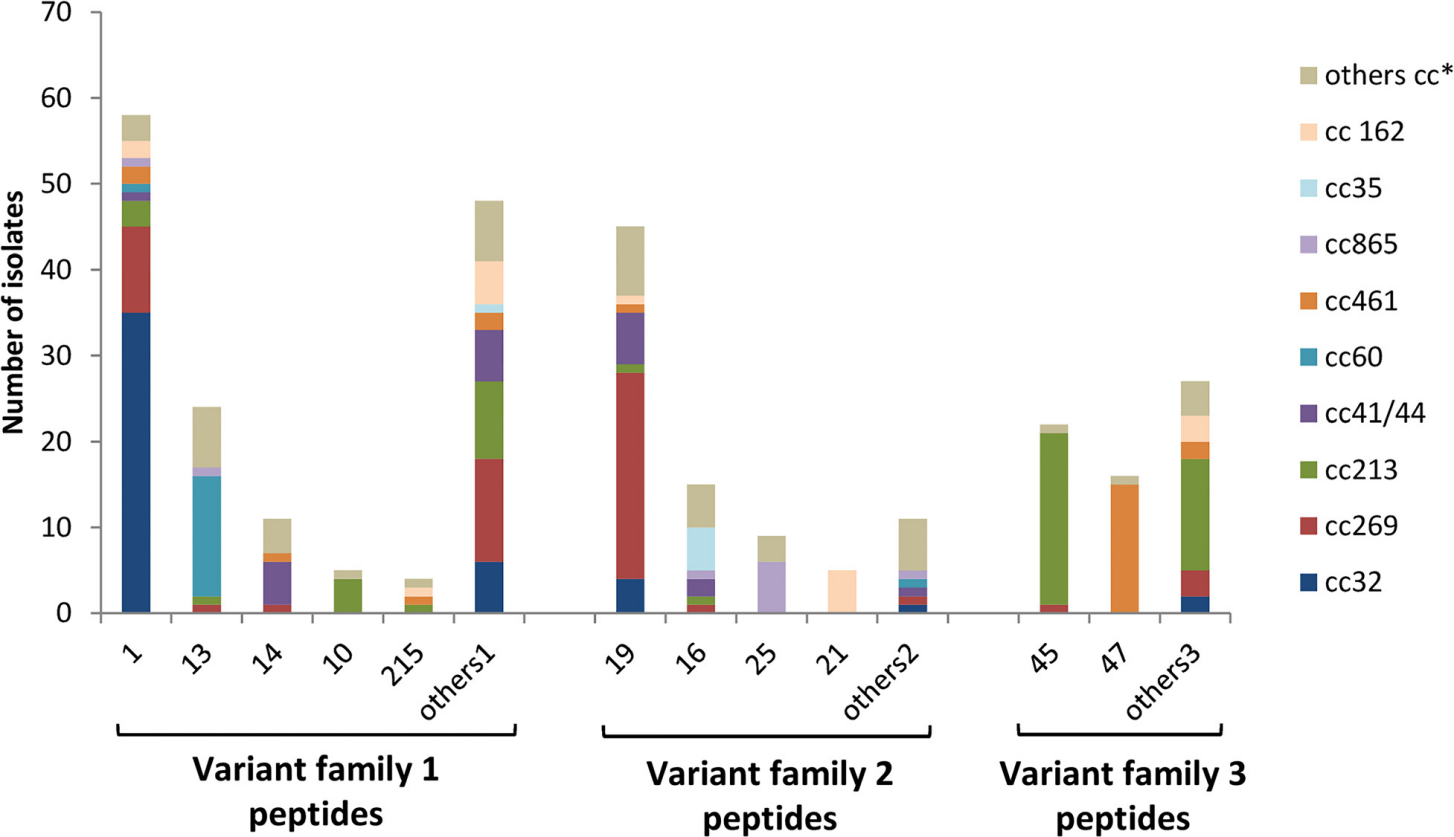
Distribution of fHbp variant families and peptides in the 300 isolates panel. Others^1^: FHbp variant family 1 peptides not present in more than three isolates (peptides 4, 15, 35, 37, 54, 65, 90, 87, 108, 110, 144, 213, 218, 236, 252,275, 322, 357, 358, 359, 360, 361, 362, 363, 365, 373, 374, 403, 456, 480, 544, 545). Others^2^: FHbp variant family 2 peptides not present in more than three isolates (peptides 18, 24, 34, 104, 367, 548, 551). Others^3^: FHbp variant family 3 peptides not present in more than three isolates (peptides 29, 31, 174, 188, 294, 364, 366, 368, 398, 399, 400,401,402, 485, 494, 532, 536, 549, 550, 552, 555). Others cc*: Others clonal complexes, included clonal complexes non assigned.

FHbp variant family 1 peptides were harboured by half of all isolates (n = 150, 50%). The most common was peptide 1 (the specific fHbp peptide included in 4CMenB/Bexsero^®^ vaccine) which was identified in 58 (19.33%) of all 300 isolates. This fHbp variant family 1 peptide 1 was mostly associated with cc32 (n = 35). FHbp variant family 2 and 3 peptides were harboured by 85 (28.33%) and 65 (21.67%) isolates, respectively. The most common fHbp variant family 2 peptide was 19 (n = 45) mostly associated with cc269 (n = 24). Peptide 45 (n = 22) was the most frequent fHbp variant family 3 peptide, and was mainly associated to cc213 (n = 20) (Fig 2).

**Fig 3:**
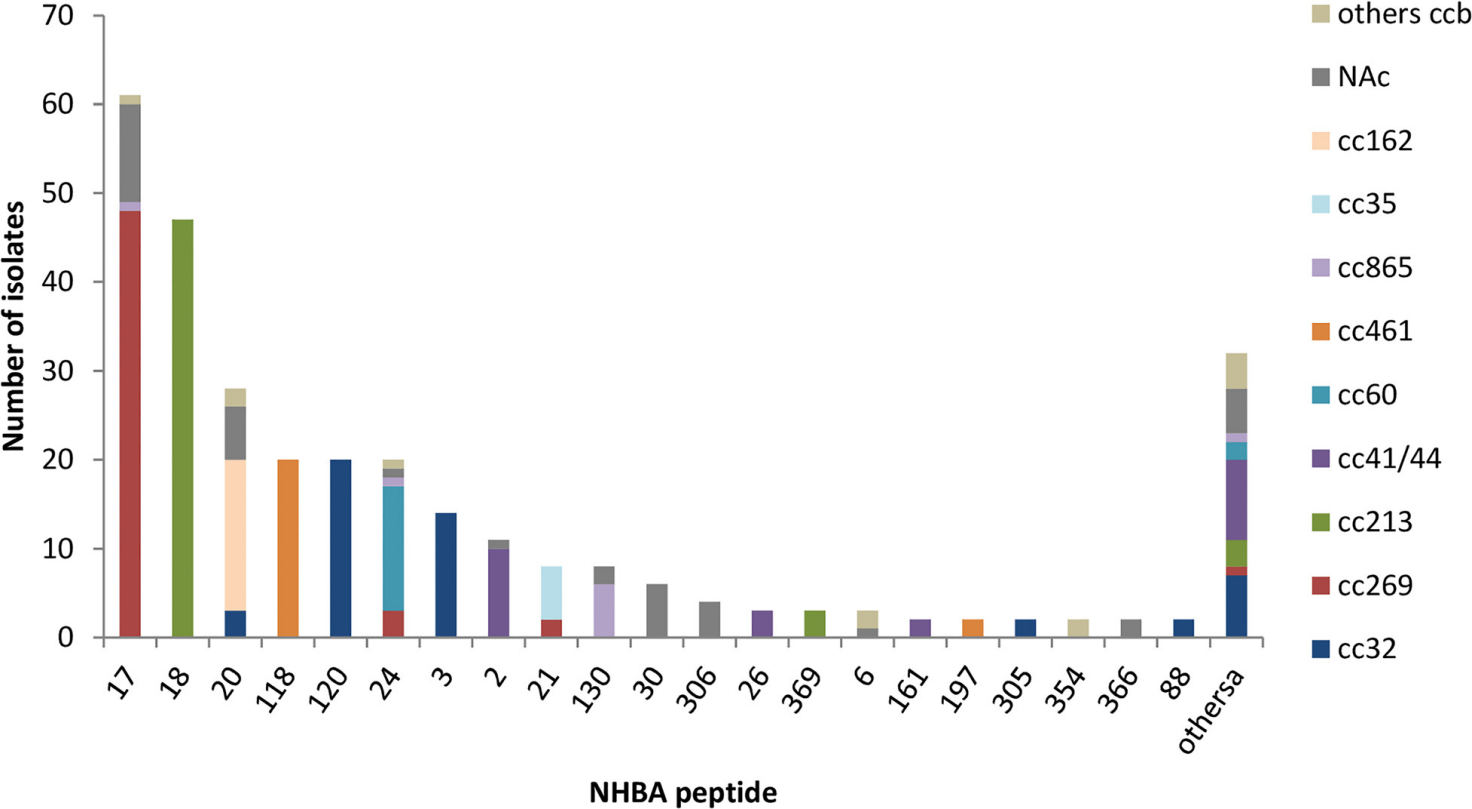
Distribution of NHBA peptides in the 300 isolates panel. Others^a^: NHBA peptides not present in more than one isolate (peptides 8, 9, 13, 25, 47, 53, 115, 160, 187, 237, 304, 307, 308, 309, 310, 355, 367, 368, 370, 385, 460, 461, 462, 463, 464, 465, 468, 469, 470, 471). Others cc^b^: Others clonal complexes. NA^c^: Clonal complexes non assigned.

All 300 *N*. *meningitidis* isolates were found to contain the *nhba* gene, however one of them presented an allele (allele 457) with a frameshift mutation (insertion of one Adenosine in 858_859 position) resulting in an internal stop codon. Fifty two different NHBA peptides were identified, of which 32 were only present in one isolate (S1 Table). The most frequent peptides were 17 (n = 61, 20.33%) and 18 (n = 47, 15.66%); rest of peptides were identified in less than 10% of isolates. Most of the strains (48 of 61) showing NHBA peptide 17 belonged to cc269, and the 47 strains showing NHBA peptide 18 belonged to cc213. The specific NHBA antigen included in the multicomponent MenB vaccine is peptide 2, and this peptide was identified in 11 (3.67%) of 300 isolates, 10 belonged to the cc41/44 and 1 without clonal complex assigned (Fig 3).

**Fig 4:**
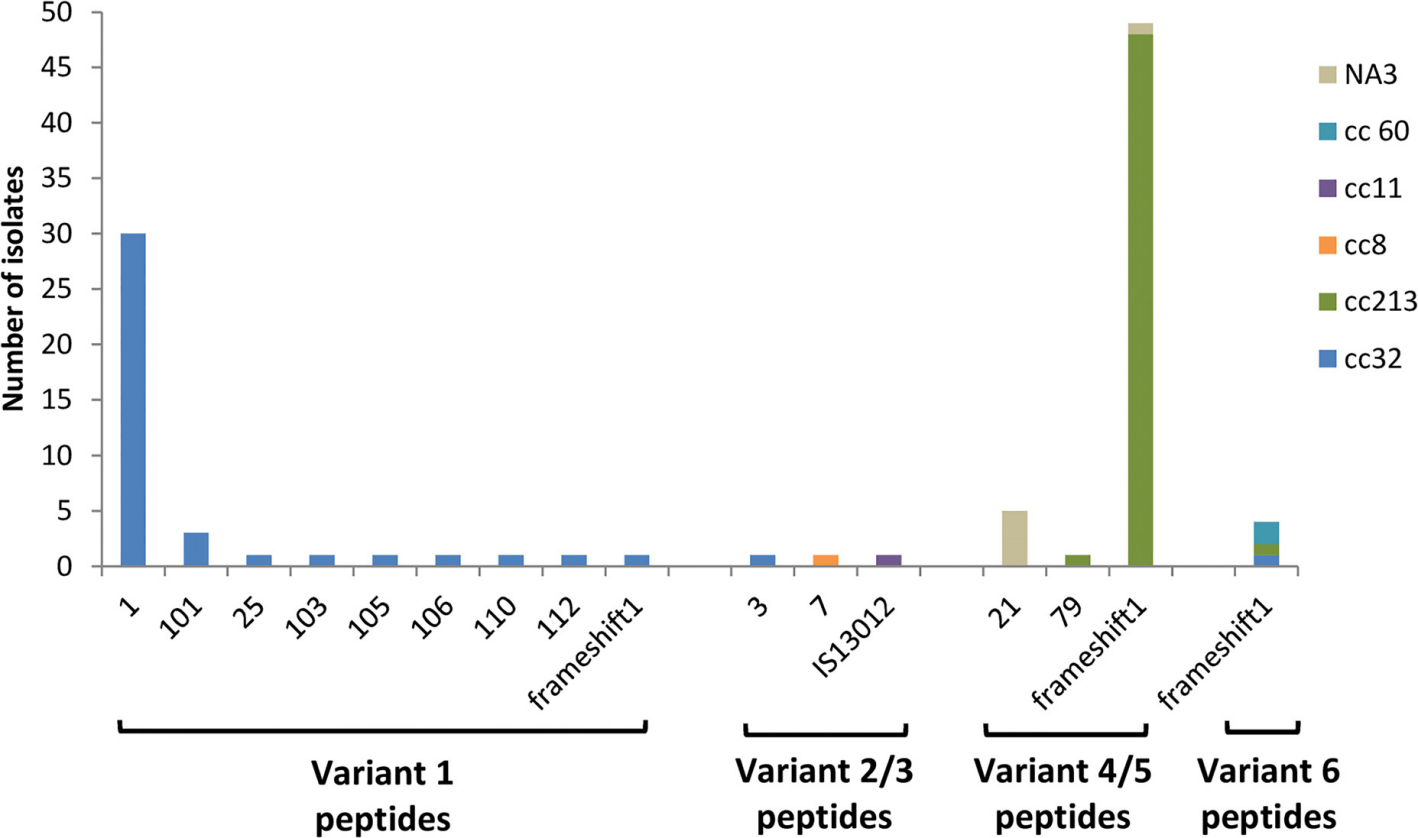
Distribution of NadA variants and peptides in the 300 isolates panel. Frameshift^1^: The *nadA* nucleotide allele presents a frameshift mutation resulting in a premature stop codon (variant 1 allele 44; variant 4/5 alleles 12, 34, 38, 39, 40 and 46; variant 6 alleles 37, 43 and 94). IS1301^2^: The *nadA* gene (allele 50) is disrupted by an insertion sequence IS1301. NA^3^: Clonal complex non assigned.

The *nadA* gene was found in 102 (34%) of the 300 isolates panel (Fig 4), but in one of them the gene was disrupted by an insertion sequence (IS1301) and 54 presented a gene with a frameshift mutation resulting in a premature stop codon. Only 47 (15.67%) isolates showed intact peptides of variant 1 (n = 39, all belonged to cc32), 2/3 (n = 2, one belonged to the cc32 and another one belonged to the cc8) or 4/5 (n = 6, one belonged to the cc213 and other 5 without cc assigned). The NadA peptide 3 of variant 2/3, included in the MenB vaccine, was only present in 1 isolate belonged to the cc32 (S1 Table).

### Meningococcal antigen typing system (MATS)

One hundred and nine (36.33%) isolates had fHbp MATS relative potencies (RPs) higher than the positive bactericidal threshold (PBT), all of them with fHbp peptides belonging to variant family 1 (peptides 1, 4, 10, 13, 14, 15, 37, 54, 69, 87, 90, 108, 110, 144, 215, 218, 236, 252, 275, 322, 358, 360, 361, 362, 365, 374, 403, 456, 480, 544, 545). The remaining 191 (63.67%) isolates, with fHbp MATS RPs lower than the PBT, had fHbp peptides belonging to variant family 1 (n = 41; peptides 10, 13, 14, 35, 37, 108, 213, 215, 236, 357, 359, 363, 373), variant family 2 (n = 85), and variant family 3 (n = 65). All strains containing fHbp variant family peptides 2 and 3 showed fHbp MATS RPs below the PBT; while strains contained fHbp variant family peptides 1 showed MATS RPs both above (n = 109) and below (n = 41) the PBT. As expected, all strains with fHbp variant family 1 peptide 1 (included in MenB vaccine) had MATS RPs higher than the PBT. Due to variations in antigen expression, differences in MATS RPs among strains with the same fHbp peptides (peptides: 10, 13, 14, 37, 108, 215 and 236) were observed.

One hundred and twenty-six (42%) isolates had NHBA MATS RPs ≥ 0.294 (NHBA PBT). Differences of MATS RPs were evident across isolates presenting the same NHBA peptide (peptides 2, 3, 6, 17, 18, 20, 21, 24, 26, 88, 118, 120, 130, 306, 354). Of the 11 strains with NHBA peptide 2 (included in MenB vaccine), 10 strains showed MATS RPs higher than the PBT and 1 lower.

Expression of NadA was above the PBT only in 4 isolates, of which 2 presented peptide 1 of variant 1, 1 strain variant 2/3 peptide 7, and other one variant 2/3 peptide 3 (the only strain with the peptide included in 4CMenB/Bexsero^®^ vaccine).

### Estimation of MenB strains coverage

The MATS results suggest that 68.67% (95% CI: 47.77–84.59%) of all isolates will be covered by the vaccine. Overall, 51.33% of isolates will be covered by one vaccine antigen (26.67% by NHBA, 22.33% by fHbp, 2% by PorA, and 0.33% by NadA), 15.33% by two antigens (10% by NHBA + fHbp, 3.33% by NHBA + PorA, 1% by fHbp + PorA, and 1% by fHbp + NadA), and 2% by three (NHBA + fHbp + PorA). No isolates will be covered by all four antigens (S1 Table).

**Fig 5:**
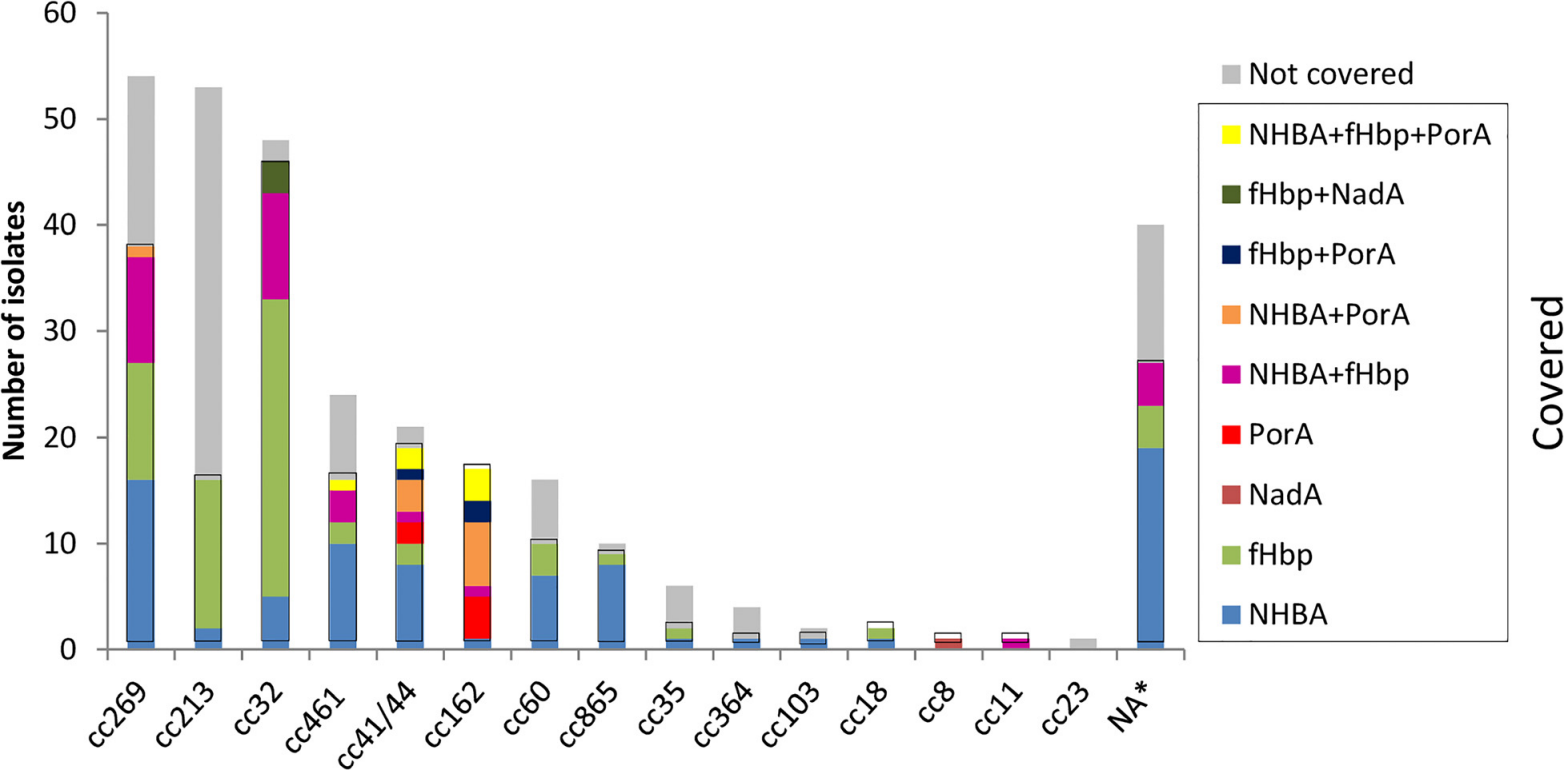
Distribution of MATS strains predicted to be covered by cc and specific vaccine antigen combinations. Strains were defined as covered if they possessed PorA P1.4 or had relative potency greater than the positive bactericidal threshold for fHbp, NHBA or NadA. *NA: Clonal complex non assigned.

The coverage within the most prevalent cc was 70.37% for the cc269, 30.19% for the cc213, and 95.83% for the cc32. The distribution of covered strains among different clonal complexes is shown in Fig 5.

## Discussion

The potential coverage of the new meningococcal B vaccine (4CMenB) can be evaluated by the MATS assay, an ELISA which measures expression and diversity of the vaccine antigens for each strain. Since the MATS value is calculated for each single antigen, it does not measures the synergism of combination of antigens, thus representing a conservative predictor of strain coverage. In a recent study [28] on a representative panel of UK MenB strains, MATS–predicted coverage (70% (55–85%) CI_0.95_) was consistently lower than coverage measured by hSBA using pooled sera from infants and adolescents (88% (72–95%) CI_0.95_). Recently additional data [29] supporting this observation were obtained from a small study in Spain. Ten strains from the overall panel of 300 MenB Spanish isolates analyzed by MATS in this study were selected for analysis in hSBA assay. In this study the 10 strains did not represent the overall Spanish panel because they were selected just among those isolates that were predicted to be not covered by MATS (with only one exception that was predicted to be covered but with RPs values close to the PBTs). All of the strains were killed by adolescents sera and 5 out of 10 strains were also killed by infants sera suggesting that the predicted coverage by MATS might be extremely conservative, particularly in adolescents, and confirming that MATS underestimates vaccine coverage.

Considering that we are using a conservative predictor for coverage and, therefore, actual vaccine effectiveness might differ from the estimates we provide, 4CMenB/Bexsero^®^ vaccine was predicted to cover the 68.67% (95% CI: 47.77–84.59%) of invasive MenB isolates in Spain. This coverage estimate is lower than in other European countries where the predicted strain coverage has been already estimated using the MATS [22, 30 31]: England and Wales (73% (57–87%) CI_0.95_), France (85% (69–93%) CI_0.95_), Germany (82% (69–93%) CI_0.95_), Norway (85% (76–98%) CI_0.95_), Italy (87% (70–93%) CI_0.95_), Greece (89.2% (63.5–99.6%) CI_0.95_) and Czech Republic (74% (59–87%) CI_0.95_). This lower Spanish coverage might be due to:

* FHbp variants family 2 and 3 peptides were harboured by 150 (50%) of 300 Spanish isolates, while in the other studied European countries this percentage ranged from 26.58% in Germany to 37.5% in France. This difference was previously observed in an earlier study [32] where systematically collected invasive MenB isolates from different European countries were characterized for *fHbp* sequence. In this case, fHbp peptides of 40.2% Spanish isolates corresponded to variants family 2 and 3, while in the other European countries ranged from 21% in Germany to 34.8% in Norway. All the analyzed strains in Spain showing fHbp peptides belonging to variants family 2 and 3 result in fHbp MATS RPs lower than the PBT. Seventy-seven of the 150 Spanish strains with fHbp variants family 2 and 3 peptides were not covered by any other of the 3 antigens included in the vaccine. Most of the 77 not covered strains belonged to cc213 and cc269 (33 and 15 strains, respectively). Clonal complexes 269 and 213 are two of the three predominant clonal complexes in Spain (18% and 19%, respectively), however these cc have little presence in most of the other European countries. The percentage of isolates belonging to the cc269 in England and Wales was 32.9% and the high prevalence of the cc269 might be associated with coverage data more similar to the Spanish data.

* A strong association between NHBA peptide 2, the specific NHBA antigen included in the 4CMenB/Bexsero^®^, and coverage with the multicomponent vaccine has been previously observed [22]. This peptide was identified only in 3.67% of the Spanish isolates, of which only one showed NHBA MATS RPs lower than the PBT. In other European countries, NHBA peptide 2 is the most frequent NHBA peptide, ranging from 31.7% in Norway to 22% in France [22]. Most of the strains showing NHBA peptide 2 belong to the cc41/44, and the prevalence of this cc was notably lower in Spain (7% vs 55.5%, 51.2%, 40.5%, 40.1%, 31.6%, 24% and 19% in Italy, Norway, France, Germany, England and Wales, Greek and Czech Republic, respectively). In contrast, NHBA peptide 18, one of the most frequent among Spanish isolates (15.67%) and little present in other countries (from 0% in Norway and Italy to 7.3% in England and Wales), has been associated with RPs lower than the PBT [22]. All the Spanish strains showing NHBA peptide 18 belong to the cc213, which has emerged in Spain during the last years. In this study only 2 of the 47 strains with NHBA peptide 18 showed NHBA MATS RPs higher than the PBT and 33 were not covered by any of the other 3 antigens included in the vaccine.

* The immunodominant protein antigen contained in the OMV-based vaccine MeNZB included in the multicomponent vaccine is PorA P1.4 serosubtype. The PorA VR2 variant 4 was identified in 8.33% of 300 Spanish isolates, elsewhere this antigen ranged in prevalence between 31.7% in Norway to 20.27% in Germany [22]. PorA VR2 4 has been found mainly associated with the cc41/44 strains in most of the European countries [22], with 187 of 213 isolates with PorA VR2 4 belonging to the cc41/44. However most of the 25 Spanish strains showing PorA VR2 4 belonged to the cc162 (15) and only 8 belonged to the cc41/44. The prevalence of both cc41/44 and cc162 was low in general in Spain.

As already mentioned, cc distribution in Spain was significantly different as compared with other countries where the predicted strains covered had been estimated using the MATS. We found three predominant cc in Spain (cc269, cc213 and cc32) while in other studied European countries (France, Germany, Norway and Italy) cc41/44 was the most prevalent cc [22]. Cc41/44 and cc269 were the predominant cc in England and Wales [22] and in Canada [33], and cc41/44 and cc32 in Czech Republic [31] and in Australia [34]. Although cc alone cannot be used to determine if an isolate is potentially covered by 4CMenB/Bexsero^®^, the cc distribution of strains between countries could account for variation in strain coverage. Countries where the most prevalent cc is cc41/44 (Italy, Norway, France and Germany) have higher coverage strain (87%, 85%, 85% and 82%, respectively) than other countries where this cc is less present (Spain, Czech Republic, Australia, England and Wales, and Canada; 68.67%, 74%, 76%, 73% and 66%, respectively) [22, 31, 33, 34]. In Greece, where cc41/44 was less present and the most frequent cc was the cc162, the overall 4CMenB/Bexsero^®^ MATS-predicted coverage was 89.2%. Cc162 is little present in the rest of studied countries, but Greek results suggest a strong association between NHBA peptide 20, presents in all cc162 strains, and predicted coverage [30]. Although cc162 is little prevalent in Spain, all strains belonging to cc162 showed NHBA peptide 20, and all were predicted to be covered by the vaccine.

Cc cannot be used to predict the vaccine antigen genotypes. In fact there were strains belonging to same cc with different antigenic profile and strains with the same antigenic variant belonging to different cc. However, we observed a strong association between some NHBA peptides and a particular cc. All strains showing NHBA peptide 18 belonged to the cc213, all NHBA 120 or NHBA 3 belonged to the cc32, all NHBA 118 belonged to the cc461, and all NHBA 2 (except one which was NA) belonged to the cc41/44. This association also has been observed in other countries [22] for NHBA 18 and 120, however the other NHBA peptides were harboured by a determinate cc but with some exceptions. In Canada [33] all strains with NHBA peptide 2 belonged to cc41/44. Further analysis with more isolates will be necessary to corroborate this association in Spain.

Isolates showing identical antigen peptides were predicted to be both, covered and not covered, confirming that is not possible to predict vaccine coverage only with the antigen genotype, neither alone nor in combination with cc. The only exception we found were the strains with fHbp peptide 1, all of which showed fHbp MATS RPs higher than the PBT. These findings agree with those obtained in other studies [22, 33].

Strains expressing multiple vaccine antigens at a level higher than the PBT are more susceptible to SBA [21], and the coverage would be maintained if one target antigen is mutated or lost the expression. The percentage of Spanish isolates covered by at least two vaccine antigens was 17.33% of all isolates which also is lower than in other studied countries (from 38% in Czech Republic to 53.6% in Germany) [22, 30, 31, 33, 34]. This finding argues for continuous detailed surveillance by MATS and genetic characterization that should allow detection of emergence of possible escape variants following the vaccine introduction.

NadA contribution to predicted strain coverage was practically nil in Spain. Although *nadA* gene was found in 102 isolates, only 4 expressed NadA with RP above the PBT. DNA sequencing of *nadA* gene in these 102 isolates revealed only 47 with intact peptides. We only found intact peptides in strains belonging to the cc32 (40 of 48 strains), the cc213 (1 of 53 strains), the cc8 (1 of 1), and non assigned cc (5 strains). The *nadA* gene was present in almost all isolates belonging to the cc213 (50 of 53 strains), all assigned to variant 4/5, but only 1 showed an intact peptide. Strains belonging to other ccs were devoid of gene *nadA*, with the exception of 2 strains belonging to cc60 (2 of 16 strains) that showed genes with a frameshift mutation resulting in a premature stop codon, and 1 belonging to cc11 (1 of 1) in which the gene was disrupted by an insertion element (IS1301). Previous studies attribute this low potential NadA coverage in MATS to the fact that NadA expression is induced in vivo while it is repressed in in-vitro conditions [22, 35]. Then, higher NadA expression levels from many of *nadA*-positive strains during active disease would be expected. However, in our study, only 47 strains showed an intact peptide, of which 43 were already covered by some of the other vaccine antigens. Of the remaining 4 strains, 2 showed NadA variant 1 peptides and 2 NadA variant 4/5 peptides. Previous studies [36] had shown absence of cross-reactivity between variant 2/3 (NadA peptide included in the vaccine is assigned to this variant) and variant 4/5, so the two strains with NadA variant 4/5 peptides would not be covered by NadA anyway. Therefore, if we consider that all *nadA*-positive strains with an intact peptide would be highly expressed and recognized, only two more strains would be covered and the predicted strain coverage would become of 69.33% instead of 68.67%. The percentage of strains covered by at least two antigens would increase from 17.33% to 26.34%, thus the chance for escape mutants to emerge with vaccine use would be lower.

This study provides a detailed breakdown of molecular characterization and MATS results for the Spanish invasive MenB strains. Because the cc distributions of strains could vary over time, which in turn could lead to changes in strain coverage, continuous detailed surveillance and monitoring of antigens expression is needed. The vaccine is already licensed in more than 30 countries worldwide, including Europe, Australia, Canada and United States, so an accurate surveillance is really important in countries where the multicomponent vaccine is being introduced. This applies especially to countries like Spain where most of the strains are predicted to be covered only by one vaccine antigen. Finally, it is important to highlight the emergence of the cc213 in Spain, which is associated with low predicted MATS strain coverage. The prevalence of the cc213 increased from being 3.6% in 2007 to 20% in 2010 [37], and therefore the incidence of this cc should monitored carefully, particularly in countries where the vaccine will be introduced.

## Supporting Information

S1 Table Distribution of sequence types (STs), PorA genotypes (PorA VR1,VR2), FetA variable regions, 4CMenB/Bexsero^®^ vaccine antigens (fHbp, NHBA, NadA) genotypes and predicted covered strains^1^ into the different clonal complexes (cc). ^1^ Strains were defined as covered by 4CMenB/Bexsero^®^ vaccine if they presented PorA P1.4 or had a RP greater than the PBT for fHbp, NHBA and/or NadA: ^a^ strains covered by NHBA (NHBA MATS RP ≥ 0.294), ^b^ strains covered by fHbp (fHbp MATS RP > 0.021), ^c^ strains covered by NadA (NadA MATS RP > 0.009), ^d^ strains covered by PorA (presence of PorA VR2 = 4), ^e^ strains covered by NHBA and fHbp, ^f^ strains covered by NHBA and PorA, ^g^ strains covered by fHbp and PorA, ^h^ strains covered by fHbp and NadA, ^i^ strains covered by NHBA, fHbp and PorA. ^2^ NA: Non assigned clonal complexes. ^3^ NadA variant/peptide -: gene not present. ^4^ FS: gene with a frameshift mutation resulting in a premature stop codon. ^5^ IS: gene disrupted by an insertion sequence (IS1301). The specific antigens included in the 4CMenB/Bexsero^®^ vaccine are labeled in red.
(DOC)
