# Supplementary material for: Predicted Strain Coverage of a New Meningococcal Multicomponent Vaccine (4CMenB) in Spain: Analysis of the Differences with Other European Countries
Source: PLoS One. 2016 Mar 7;11(3):e0150721. doi: 10.1371/journal.pone.0150721 (PMC4780694; doi:10.1371/journal.pone.0150721)
Supplement: S1 Table — 1 Strains were defined as covered by 4CMenB/Bexsero® vaccine if they presented PorA P1.4 or had a RP greater than the PBT for fHbp, NHBA and/or NadA: a strains covered by NHBA (NHBA MATS RP ≥ 0.294), b strains covered by fHbp (fHbp MATS RP > 0.021), c strains covered by NadA (NadA MATS RP > 0.009), d strains covered by PorA (presence of PorA VR2 = 4), e strains covered by NHBA and fHbp, f strains covered by NHBA and PorA, g strains covered by fHbp and PorA, h strains covered by fHbp and NadA, i strains covered by NHBA, fHbp and PorA. 2 NA: Non assigned clonal complexes. 3 NadA variant/peptide -: gene not present. 4 FS: gene with a frameshift mutation resulting in a premature stop codon. 5 IS: gene disrupted by an insertion sequence (IS1301). The specific antigens included in the 4CMenB/Bexsero® vaccine are labeled in red. (DOC) [file pone.0150721.s001.doc]

**S1 Table. Distribution of sequence types (STs), PorA genotypes (PorA VR1,VR2), FetA variable regions, 4CMenB/Bexsero® vaccine antigens (fHbp, NHBA, NadA) genotypes and predicted covered strains^1^** into the different clonal complexes (cc).

| **cc 269 (n)** | | | | | | | | | |
| --- | --- | --- | --- | --- | --- | --- | --- | --- | --- |
| **ST** | **PorA VR1, VR2** | **FetA** | **fHbp** | | **NHBA** | **NadA** | | **N^o^ of covered strains^1^** | **N^o^ of not covered strains** |
|  |  |  | **Variant family** | **Peptide** | **Peptide** | **Variant** | **Peptide** |  |  |
| ST-1163 (34) | 22,9 (30) | F1-5 (1) | 2 (1) | 19 (1) | 17 (1) | -^3^ (1) | -^3^ (1) | 1^a^ | 0 |
|  |  | F1-7 (3) | 2 (2) | 19 (2) | 17 (2) | - (2) | - (2) | 0 | 2 |
|  |  |  | 1 (1) | 361 (1) | 17 (1) | - (1) | - (1) | 1^b^ | 0 |
|  |  | F1-55 (8) | 1 (3) | 362 (2) | 17 (2) | - (2) | - (2) | 2^e^ | 0 |
|  |  |  |  | 14 (1) | 17 (1) | - (1) | - (1) | 1^b^ | 0 |
|  |  |  | 2 (4) | 19 (4) | 17 (4) | - (4) | - (4) | 1^a^ | 3 |
|  |  |  | 3 (1) | 552 (1) | 17 (1) | - (1) | - (1) | 1^a^ | 0 |
|  |  | F3-7 (2) | 1 (1) | 357 (1) | 17 (1) | - (1) | - (1) | 0 | 1 |
|  |  |  | 3 (1) | 45 (1) | 17 (1) | - (1) | - (1) | 1^a^ | 0 |
|  |  | F5-1 (1) | 1 (1) | 1 (1) | 17 (1) | - (1) | - (1) | 1^b^ | 0 |
|  |  | F5-2 (1) | 3 (1) | 555 (1) | 17 (1) | - (1) | - (1) | 0 | 1 |
|  |  | F5-5 (1) | 1 (1) | 1 (1) | 17 (1) | - (1) | - (1) | 1^b^ | 0 |
|  |  | F5-12 (13) | 1 (5) | 1 (2) | 17 (2) | - (2) | - (2) | 2^b^ | 0 |
|  |  |  |  | 13 (1) | 17 (1) | - (1) | - (1) | 1^a^ | 0 |
|  |  |  |  | 359 (1) | 17 (1) | - (1) | - (1) | 1^a^ | 0 |
|  |  |  |  | 480 (1) | 17 (1) | - (1) | - (1) | 1^e^ | 0 |
|  |  |  | 2 (8) | 19 (7) | 17 (6) | - (6) | - (6) | 5^a^ | 1 |
|  |  |  |  |  | 24 (1) | - (1) | - (1) | 1^a^ | 0 |
|  |  |  |  | 16 (1) | 17 (1) | - (1) | - (1) | 1^a^ | 0 |
|  | 22,10 (1) | F3-6 (1) | 2 (1) | 19 (1) | 17 (1) | - (1) | - (1) | 1^a^ | 0 |
|  | 22,14 (1) | F4-1 (1) | 1 (1) | 360 (1) | 17 (1) | - (1) | - (1) | 1^e^ | 0 |
|  | 7,9 (1) | F1-55 (1) | 1 (1) | 1 (1) | 385 (1) | - (1) | - (1) | 1^b^ | 0 |
|  | 19,15(1) | F5-12 (1) | 2 (1) | 19 (1) | 17 (1) | - (1) | - (1) | 0 | 1 |
| ST-6416 (5) | 22,9 (5) | F1-55 (5) | 1 (2) | 90 (2) | 17 (2) | - (2) | - (2) | 2^b(1),e(1)^ | 0 |
|  |  |  | 2 (3) | 19 (3) | 17 (3) | - (3) | - (3) | 0 | 3 |
| ST-8748 (5) | 22,9 (5) | F1-55 (4) | 1 (3) | 1 (3) | 17 (3) | - (3) | - (3) | 3^b(1),e(2)^ | 0 |
|  |  |  | 2 (1) | 19 (1) | 17 (1) | - (1) | - (1) | 0 | 1 |
|  |  | F5-9 (1) | 1 (1) | 1 (1) | 17 (1) | - (1) | - (1) | 1^e^ | 0 |
| ST-7981 (2) | 22,9 (2) | F1-7 (1) | 2 (1) | 19 (1) | 17 (1) | - (1) | - (1) | 0 | 1 |
|  |  | F1-55 (1) | 3 (1) | 366 (1) | 17 (1) | - (1) | - (1) | 0 | 1 |
| ST-2561 (1) | 22,9 (1) | F5-12 (1) | 2 (1) | 367 (1) | 24 (1) | - (1) | - (1) | 1^a^ | 0 |
| ST-467 (1) | 18-1,3 (1) | F1-7 (1) | 1 (1) | 15 (1) | 21 (1) | - (1) | - (1) | 1^b^ | 0 |
| ST-6609 (1) | 22,9 (1) | F5-12 (1) | 2 (1) | 19 (1) | 17 (1) | - (1) | - (1) | 0 | 1 |
| ST-8746 (1) | 22,9 (1) | F1-55 (1) | 2 (1) | 19 (1) | 17 (1) | - (1) | - (1) | 1^a^ | 0 |
| ST-8827 (1) | 22,9 (1) | F1-55 (1) | 1 (1) | 1 (1) | 24 (1) | - (1) | - (1) | 1^b^ | 0 |
| ST-8872 (1) | 21,4 (1) | F1-7 (1) | 2 (1) | 19 (1) | 17 (1) | - (1) | - (1) | 1^f^ | 0 |
| ST-8938 (1) | 19-1,15 (1) | F5-5 (1) | 1 (1) | 15 (1) | 21 (1) | - (1) | - (1) | 1^e^ | 0 |
| ST-8952 (1) | 22,9 (1) | F5-1 (1) | 1 (1) | 403 (1) | 17 (1) | - (1) | - (1) | 1^e^ | 0 |
| **TOTAL** | **54** | | | | | | | **38** | **16** |

| **cc 213 (n)** | | | | | | | | | |
| --- | --- | --- | --- | --- | --- | --- | --- | --- | --- |
| **ST** | **PorA VR1, VR2** | **FetA** | **fHbp** | | **NHBA** | **NadA** | | **N^o^ of covered strains** | **N^o^ of not covered strains** |
|  |  |  | **Variant family** | **Peptide** | **Peptide** | **Variant** | **Peptide** |  |  |
| ST-3496 (18) | 22,14 (18) | F1-5 (1) | 3 (1) | 401 (1) | 18 (1) | 4/5 (1) | FS^4^ (1) | 0 | 1 |
|  |  | F2-9 (1) | 3 (1) | 45 (1) | 53 (1) | 4/5 (1) | FS (1) | 0 | 1 |
|  |  | F3-6 (2) | 1 (1) | 10 (1) | 369 (1) | 4/5 (1) | FS (1) | 1^b^ | 0 |
|  |  |  | 3 (1) | 29 (1) | 18 (1) | 4/5 (1) | FS (1) | 0 | 1 |
|  |  | F5-2 (1) | 1 (1) | 374 (1) | 18 (1) | 4/5 (1) | FS (1) | 1^b^ | 0 |
|  |  | F5-5 (12) | 1 (5) | 1 (2) | 18 (2) | 4/5 (2) | FS (2) | 2^b^ | 0 |
|  |  |  |  | 10 (1) | 369 (1) | 4/5 (1) | FS (1) | 0 | 1 |
|  |  |  |  | 13 (1) | 18 (1) | 4/5 (1) | FS (1) | 0 | 1 |
|  |  |  |  | 215 (1) | 18 (1) | 6 (1) | FS (1) | 0 | 1 |
|  |  |  | 2 (2) | 16 (1) | 18 (1) | 4/5 (1) | FS (1) | 1^a^ | 0 |
|  |  |  |  | 19 (1) | 18 (1) | 4/5 (1) | FS (1) | 0 | 1 |
|  |  |  | 3 (5) | 45 (3) | 18 (3) | 4/5 (3) | FS (3) | 0 | 3 |
|  |  |  |  | 188 (1) | 18 (1) | 4/5 (1) | FS (1) | 0 | 1 |
|  |  |  |  | 400 (1) | 18 (1) | 4/5 (1) | FS (1) | 0 | 1 |
|  |  | F5-9 (1) | 1 (1) | 236 (1) | 18 (1) | 4/5 (1) | FS (1) | 1^b^ | 0 |
| ST-213 (16) | 22,14 (14) | F1-5 (1) | 3 (1) | 45 (1) | 18 (1) | 4/5 (1) | FS (1) | 0 | 1 |
|  |  | F5-5 (13) | 1 (1) | 213 (1) | 18 (1) | 4/5 (1) | FS (1) | 0 | 1 |
|  |  |  | 3 (12) | 45 (8) | 18 (7) | 4/5 (7) | FS (7) | 1^a^ | 6 |
|  |  |  |  |  | 115 (1) | - (1) | - (1) | 0 | 1 |
|  |  |  |  | 294 (1) | 18 (1) | 4/5 (1) | FS (1) | 0 | 1 |
|  |  |  |  | 364 (1) | 18 (1) | 4/5 (1) | FS (1) | 0 | 1 |
|  |  |  |  | 402 (1) | 18 (1) | 4/5 (1) | FS (1) | 0 | 1 |
|  |  |  |  | 494 (1) | 18 (1) | 4/5 (1) | FS (1) | 0 | 1 |
|  | 22,2 (1) | F5-8 (1) | 3 (1) | 368 (1) | 18 (1) | 4/5 (1) | FS (1) | 0 | 1 |
|  | 22-29,14 (1) | F1-5 (1) | 3 (1) | 45 (1) | 18 (1) | 4/5 (1) | FS (1) | 0 | 1 |
| ST-8750 (4) | 22,14 (4) | F5-5 (4) | 1 (1) | 218 (1) | 18 (1) | 4/5 (1) | FS (1) | 1^b^ | 0 |
|  |  |  | 3 (3) | 45 (3) | 18 (3) | 4/5 (3) | FS (3) | 0 | 3 |
| ST-7231 (2) | 22,14 (2) | F5-9 (2) | 1 (2) | 275 (2) | 18 (2) | 4/5 (2) | FS (2) | 2^b^ | 0 |
| ST-2391(1) | 22,14 (1) | F5-100 (1) | 3 (1) | 45 (1) | 18 (1) | 4/5 (1) | FS (1) | 0 | 1 |
| ST-2660 (1) | 22,14 (1) | F1-7 (1) | 3 (1) | 45 (1) | 460 (1) | 4/5 (1) | FS (1) | 0 | 1 |
| ST-8071 (1) | 22,14 (1) | F3-9 (1) | 3 (1) | 45 (1) | 18 (1) | 4/5 (1) | FS (1) | 0 | 1 |
| ST-8743 (1) | 22,14 (1) | F5-5 (1) | 3 (1) | 398 (1) | 18 (1) | 4/5 (1) | FS (1) | 0 | 1 |
| ST-8756 (1) | 22,14 (1) | F5-5 (1) | 1 (1) | 110 (1) | 18 (1) | 4/5 (1) | FS (1) | 1^b^ | 0 |
| ST-8826 (1) | 22,14 (1) | F5-5 (1) | 1 (1) | 69 (1) | 18 (1) | 4/5 (1) | FS (1) | 1^b^ | 0 |
| ST-11220 (1) | 22,14 (1) | F5-5(1) | 1 (1) | 10 (1) | 18 (1) | 4/5 (1) | FS (1) | 1^b^ | 0 |
| ST-8835 (1) | 22,14 (1) | F5-12 (1) | 1 (1) | 10 (1) | 369 (1) | 4/5 (1) | 79 (1) | 1^b^ | 0 |
| ST-8840 (1) | 22,14 (1) | F5-5 (1) | 3 (1) | 550 (1) | 18 (1) | - (1) | - (1) | 0 | 1 |
| ST-8862 (1) | 22,14 (1) | F5-5 (1) | 1 (1) | 1 (1) | 18 (1) | 4/5 (1) | FS (1) | 1^b^ | 0 |
| ST-8892 (1) | 22,14 (1) | F3-6 (1) | 3 (1) | 485 (1) | 18 (1) | 4/5 (1) | FS (1) | 0 | 1 |
| ST-8954 (1) | 22,14 (1) | F5-5 (1) | 1 (1) | 456 (1) | 18 (1) | 4/5 (1) | FS (1) | 1^b^ | 0 |
| ST-8955 (1) | 22,14 (1) | F4-1 (1) | 3 (1) | 549 (1) | 18 (1) | - (1) | - (1) | 0 | 1 |
| **TOTAL** | **53** | | | | | | | **16** | **37** |

| **cc 32 (n)** | | | | | | | | | |
| --- | --- | --- | --- | --- | --- | --- | --- | --- | --- |
| **ST** | **PorA VR1, VR2** | **FetA** | **fHbp** | | **NHBA** | **NadA** | | **N^o^ of covered strains** | **N^o^ of not covered strains** |
|  |  |  | **Variant family** | **Peptide** | **Peptide** | **Variant** | **Peptide** |  |  |
| ST-749 (19) | 7,15 (3) | F5-1 (3) | 1 (2) | 1 (2) | 120 (2) | 1 (1) | 1 (1) | 1^b^ | 0 |
|  |  |  |  |  |  | - (1) | - (1) | 1^b^ | 0 |
|  |  |  | 2 (1) | 19 (1) | 120 (1) | 1 (1) | 1 (1) | 1^a^ | 0 |
|  | 19,15 (16) | F1-5 (1) | 1 (1) | 1 (1) | 120 (1) | 1 (1) | 1 (1) | 1^b^ | 0 |
|  |  | F1-7 (1) | 1 (1) | 1 (1) | 120 (1) | - (1) | - (1) | 1^b^ | 0 |
|  |  | F1-55 (1) | 1 (1) | 1 (1) | 120 (1) | 1 (1) | 1 (1) | 1^b^ | 0 |
|  |  | F5-1 (12) | 1 (10) | 1 (9) | 120 (9) | 1 (8) | 1(7) | 7^b(6),h(1)^ | 0 |
|  |  |  |  |  |  |  | 106 (1) | 1^b^ | 0 |
|  |  |  |  |  |  | - (1) | - (1) | 1^b^ | 0 |
|  |  |  |  | 358 (1) | 120 (1) | 1 (1) | 1 (1) | 1^b^ | 0 |
|  |  |  | 2 (1) | 19 (1) | 120 (1) | 1 (1) | 105 (1) | 0 | 1 |
|  |  |  | 3 (1) | 401 (1) | 120 (1) | 1 (1) | 1 (1) | 0 | 1 |
|  |  | F5-5 (1) | 1 (1) | 1 (1) | 120 (1) | 1 (1) | 1 (1) | 1^b^ | 0 |
| ST-32 (7) | 7-2,16 (7) | F3-3 (7) | 1 (7) | 1 (7) | 3 (3) | 1 (3) | 1 (3) | 3^b(1),e(1),h(1)^ | 0 |
|  |  |  |  |  | 88 (2) | 1 (2) | 101 (2) | 2^b(1),e(1)^ | 0 |
|  |  |  |  |  | 305 (1) | 1 (1) | 101 (1) | 1^b^ | 0 |
|  |  |  |  |  | 462 (1) | 1 (1) | 1 (1) | 1^b^ | 0 |
| ST-33 (7) | 19,15 (5) | F1-106 (1) | 1 (1) | 1 (1) | 3 (1) | - (1) | - (1) | 1^e^ | 0 |
|  |  | F5-1 (4) | 1 (3) | 1 (3) | 3 (3) | 1 (2) | 1 (1) | 1^e^ | 0 |
|  |  |  |  |  |  |  | 25 (1) | 1^e^ | 0 |
|  |  |  |  |  |  | 6 (1) | FS (1) | 1^b^ | 0 |
|  |  |  | 2 (1) | 19 (1) | 3 (1) | 1 (1) | 1 (1) | 1^a^ | 0 |
|  | 5,2-7 (2) | F3-6 (2) | 1 (2) | 144 (2) | 20 (2) | 1 (2) | 1 (2) | 2^b(1),e(1)^ | 0 |
| ST-7024 (2) | 19,15 (2) | F1-29 (1) | 1 (1) | 1 (1) | 305 (1) | 1 (1) | 112 (1) | 1^b^ | 0 |
|  |  | F5-13 (1) | 2 (1) | 104 (1) | 309 (1) | 1 (1) | 1 (1) | 1^a^ | 0 |
| ST-1618 (1) | 19,15 (1) | F5-1 (1) | 1 (1) | 1 (1) | 3 (1) | 1 (1) | 1 (1) | 1^b^ | 0 |
| ST-6589 (1) | 19,15 (1) | F5-12 (1) | 1 (1) | 1 (1) | 3 (1) | 1 (1) | 1 (1) | 1^e^ | 0 |
| ST-7003 (1) | 19,15 (1) | F5-1 (1) | 1 (1) | 1 (1) | 464 (1) | - (1) | - (1) | 1^e^ | 0 |
| ST-7312 (1) | 19,15 (1) | F1-96 (1) | 1 (1) | 1 (1) | 3 (1) | 1 (1) | 1 (1) | 1^b^ | 0 |
| ST-800 (1) | 7,16 (1) | F3-3 (1) | 1 (1) | 1 (1) | 3 (1) | 1 (1) | 1 (1) | 1^e^ | 0 |
| ST-8745 (1) | 19,15 (1) | F4-28 (1) | 1 (1) | 1 (1) | FS (1) | 1 (1) | 1 (1) | 1^b^ | 0 |
| ST-8758 (1) | 19,15 (1) | F5-1 (1) | 1 (1) | 218 (1) | 3 (1) | 1 (1) | 103 (1) | 1^b^ | 0 |
| ST-34 (1) | 19,15 (1) | F4-28 (1) | 1 (1) | 1 (1) | 187 (1) | 1 (1) | 1 (1) | 1^b^ | 0 |
| ST-8864 (1) | 19,15 (1) | F5-1 (1) | 1 (1) | 545 (1) | 120 (1) | 2/3 (1) | 3 (1) | 1^h^ | 0 |
| ST-8868 (1) | 7,16 (1) | F3-7 (1) | 3 (1) | 494 (1) | 237 (1) | 1 (1) | 110 (1) | 1^a^ | 0 |
| ST-8956 (1) | 19,15 (1) | F5-1 (1) | 2 (1) | 19 (1) | 463 (1) | 1 (1) | FS (1) | 1^a^ | 0 |
| ST-8957 (1) | 5,2 (1) | F5-1 (1) | 1 (1) | 144 (1) | 20 (1) | - (1) | - (1) | 1^b^ | 0 |
| ST-9266 (1) | 7-2,16 (1) | F5-5 (1) | 1 (1) | 1 (1) | 3 (1) | 1 (1) | 1 (1) | 1^e^ | 0 |
| **TOTAL** | **48** | | | | | | | **46** | **2** |

| **cc 461 (n)** | | | | | | | | | |
| --- | --- | --- | --- | --- | --- | --- | --- | --- | --- |
| **ST** | **PorA VR1, VR2** | **FetA** | **fHbp** | | **NHBA** | **NadA** | | **N^o^ of covered strains** | **N^o^ of not covered strains** |
|  |  |  | **Variant family** | **Peptide** | **Peptide** | **Variant** | **Peptide** |  |  |
| ST-461 (8) | 5-1,10-1 (1) | F3-9 (1) | 3 (1) | 47 (1) | 197 (1) | - (1) | - (1) | 0 | 1 |
|  | 17,9 (1) | F5-5 (1) | 3 (1) | 47 (1) | 118 (1) | - (1) | - (1) | 1^a^ | 0 |
|  | 19-2,13 (1) | F1-7 (1) | 1 (1) | 1 (1) | 118 (1) | - (1) | - (1) | 1^b^ | 0 |
|  | 19-2,13-1 (3) | F1-7 (1) | 3 (1) | 47 (1) | 118 (1) | - (1) | - (1) | 1^a^ | 0 |
|  |  | F5-5 (1) | 3 (1) | 47 (1) | 549 (1) | - (1) | - (1) | 0 | 1 |
|  |  | F3-9 (1) | 1 (1) | 544 (1) | 118 (1) | - (1) | - (1) | 1^e^ | 0 |
|  | 19-2,13-2 (1) | F5-5 (1) | 3 (1) | 47 (1) | 368 (1) | - (1) | - (1) | 0 | 1 |
|  | 22,14 (1) | F5-9 (1) | 3 (1) | 174 (1) | 118 (1) | - (1) | - (1) | 0 | 1 |
| ST-1946 (7) | 5-1,deleted (1) | F3-9 (1) | 3 (1) | 399 (1) | 197 (1) | - (1) | - (1) | 0 | 1 |
|  | 18-1,3 (2) | F3-9 (1)  F5-8 (1) | 2 (1) | 19 (1) | 118 (1) | - (1) | - (1) | 1^a^ | 0 |
|  |  |  | 3 (1) | 47 (1) | 118 (1) | - (1) | - (1) | 1^a^ | 0 |
|  | 19-1,13-1 (1) | F3-9 (1) | 3 (1) | 47 (1) | 118 (1) | - (1) | - (1) | 1^a^ | 0 |
|  | 19-2,13 (1) | F3-9 (1) | 3 (1) | 47 (1) | 118 (1) | - (1) | - (1) | 1^a^ | 0 |
|  | 19-2,13-1 (1) | F3-9 (1) | 3 (1) | 47 (1) | 118 (1) | - (1) | - (1) | 1^a^ | 0 |
|  | 19-2,13-15 (1) | F3-9 (1) | 3 (1) | 47 (1) | 118 (1) | - (1) | - (1) | 0 | 1 |
| ST-4775 (1) | 19-2,13 (1) | F5-5 (1) | 3 (1) | 47 (1) | 118 (1) | - (1) | - (1) | 1^a^ | 0 |
| ST-7010 (1) | 19-2,13-2 (1) | F5-5 (1) | 3 (1) | 47 (1) | 310 (1) | - (1) | - (1) | 0 | 1 |
| ST-8873 (1) | 19-2,4 (1) | F3-9 (1) | 1 (1) | 322 (1) | 118 (1) | - (1) | - (1) | 1^i^ | 0 |
| ST-8744 (1) | 19-2,13-1 (1) | F3-9 (1) | 3 (1) | 47 (1) | 118 (1) | - (1) | - (1) | 1^a^ | 0 |
| ST-8859 (1) | 19-2,13-1 (1) | F3-9 (1) | 1 (1) | 1 (1) | 118 (1) | - (1) | - (1) | 1^b^ | 0 |
| ST-8861 (1) | 19-2,13(1) | F3-9 (1) | 1 (1) | 14 (1) | 118 (1) | - (1) | - (1) | 1^e^ | 0 |
| ST-9264 (1) | 18-1,3 (1) | F1-7 (1) | 3 (1) | 47 (1) | 118 (1) | - (1) | - (1) | 1^a^ | 0 |
| ST-9267 (1) | 22,9 (1) | F3-9 (1) | 1 (1) | 215 (1) | 118 (1) | - (1) | - (1) | 1^e^ | 0 |
| ST-9291 (1) | 7,30-3 (1) | F5-5 (1) | 3 (1) | 47 (1) | 118 (1) | - (1) | - (1) | 0 | 1 |
| **TOTAL** | **24** | | | | | | | **16** | **8** |

| **cc 41/44 (n)** | | | | | | | | | |
| --- | --- | --- | --- | --- | --- | --- | --- | --- | --- |
| **ST** | **PorA VR1, VR2** | **FetA** | **fHbp** | | **NHBA** | **NadA** | | **N^o^ of covered strains** | **N^o^ of not covered strains** |
|  |  |  | **Variant family** | **Peptide** | **Peptide** | **Variant** | **Peptide** |  |  |
| ST-41 (4) | 7-2,4 (4) | F5-17 (2) | 1 (2) | 363 (2) | 26 (2) | - (2) | - (2) | 2^d(1),f(1)^ | 0 |
|  |  | F1-5 (2) | 1 (2) | 4 (1) | 2 (1) | - (1) | - (1) | 1^i^ | 0 |
|  |  |  |  | 35 (1) | 26 (1) | - (1) | - (1) | 1^d^ | 0 |
| ST-1947 (2) | 7-2,14 (1) | F1-7 (1) | 2 (1) | 16 (1) | 161 (1) | - (1) | - (1) | 1^a^ | 0 |
|  | 7-2,14-34 (1) | F1-7 (1) | 2 (1) | 16 (1) | 161 (1) | - (1) | - (1) | 1^a^ | 0 |
| ST-3754 (2) | 12-1,13-1 (1) | F4-17 (1) | 1 (1) | 1 (1) | 2 (1) | - (1) | - (1) | 1^b^ | 0 |
|  | 18-1,3 (1) | F1-5 (1) | 1 (1) | 14 (1) | 47 (1) | - (1) | - (1) | 1^b^ | 0 |
| ST-414 (2) | 5-2,10-2 (1) | F1-5 (1) | 2 (1) | 19 (1) | 461 (1) | - (1) | - (1) | 1^a^ | 0 |
|  | 18,25-11 (1) | F5-2 (1) | 2 (1) | 19 (1) | 2 (1) | - (1) | - (1) | 1^a^ | 0 |
| ST-1403 (1) | 7-2,4 (1) | Not amplified (1) | 1 (1) | 14 (1) | 160 (1) | - (1) | - (1) | 1^g^ | 0 |
| ST-1851 (1) | 7-2,4 (1) | F5-8 (1) | 1 (1) | 14 (1) | 2 (1) | - (1) | - (1) | 1^f^ | 0 |
| ST-191 (1) | 21,16 (1) | F1-55 (1) | 2 (1) | 24 (1) | 9 (1) | - (1) | - (1) | 0 | 1 |
| ST-409 (1) | 18-1,34 (1) | F1-5 (1) | 2 (1) | 19 (1) | 2 (1) | - (1) | - (1) | 1^a^ | 0 |
| ST-437 (1) | 19-22,26 (1) | F5-2 (1) | 2 (1) | 19 (1) | 308 (1) | - (1) | - (1) | 0 | 1 |
| ST-6744 (1) | 7-2,4 (1) | F3-6 (1) | 1 (1) | 14 (1) | 471 (1) | - (1) | - (1) | 1^f^ | 0 |
| ST-7188 (1) | 18,25 (1) | F1-5 (1) | 1 (1) | 236 (1) | 2 (1) | - (1) | - (1) | 1^a^ | 0 |
| ST-8751 (1) | 18-1,34 (1) | F1-5 (1) | 1 (1) | 15 (1) | 2 (1) | - (1) | - (1) | 1^e^ | 0 |
| ST-8825 (1) | 7-2,4 (1) | F1-5 (1) | 1 (1) | 14 (1) | 2 (1) | - (1) | - (1) | 1^i^ | 0 |
| ST-8863 (1) | 18-1,34 (1) | F1-5 (1) | 2 (1) | 19 (1) | 2 (1) | - (1) | - (1) | 1^a^ | 0 |
| ST-8869 (1) | 18-1,34 (1) | F5-1 (1) | 2 (1) | 19 (1) | 2 (1) | - (1) | - (1) | 1^a^ | 0 |
| **TOTAL** | **21** | | | | | | | **19** | **2** |

| **cc 162 (n)** | | | | | | | | | |
| --- | --- | --- | --- | --- | --- | --- | --- | --- | --- |
| **ST** | **PorA VR1, VR2** | **FetA** | **fHbp** | | **NHBA** | **NadA** | | **N^o^ of covered strains** | **N^o^ of not covered strains** |
|  |  |  | **Variant family** | **Peptide** | **Peptide** | **Variant** | **Peptide** |  |  |
| ST-162 (11) | 7-2,4 (11) | F5-9 (10) | 1 (6) | 1 (2) | 20 (2) | - (2) | - (2) | 2^g(1),i(1)^ | 0 |
|  |  |  |  | 87 (1) | 20 (1) | - (1) | - (1) | 1^i^ | 0 |
|  |  |  |  | 213 (1) | 20 (1) | - (1) | - (1) | 1^f^ | 0 |
|  |  |  |  | 359 (1) | 20 (1) | - (1) | - (1) | 1^f^ | 0 |
|  |  |  |  | 365 (1) | 20 (1) | - (1) | - (1) | 1^f^ | 0 |
|  |  |  | 2 (3) | 21 (3) | 20 (3) | - (3) | - (3) | 3^d(2),f(1)^ | 0 |
|  |  |  | 3 (1) | 536 (1) | 20 (1) | - (1) | - (1) | 1^d^ | 0 |
|  |  | F1-7 (1) | 2 (1) | 21 (1) | 20 (1) | - (1) | - (1) | 1^f^ | 0 |
| ST-1240 (1) | 7-2,4 (1) | F5-9 (1) | 3 (1) | 174 (1) | 20 (1) | - (1) | - (1) | 1^d^ | 0 |
| ST-7247 (1) | 7-2,4 (1) | F5-9 (1) | 3 (1) | 536 (1) | 20 (1) | - (1) | - (1) | 1^f^ | 0 |
| ST-752 (1) | 22,14 (1) | F5-9 (1) | 2 (1) | 19 (1) | 20 (1) | - (1) | - (1) | 1^a^ | 0 |
| ST-8824 (1) | 7-2,4 (1) | F5-9 (1) | 1 (1) | 54 (1) | 20 (1) | - (1) | - (1) | 1^i^ | 0 |
| ST-8839 (1) | 7-2,4 (1) | F5-8 (1) | 2 (1) | 21 (1) | 20 (1) | - (1) | - (1) | 1^f^ | 0 |
| ST-8891 (1) | 22,14 (1) | F5-9 (1) | 1 (1) | 215 (1) | 20 (1) | - (1) | - (1) | 1^e^ | 0 |
| **TOTAL** | **17** | | | | | | | **17** | **0** |

| **cc 60 (n)** | | | | | | | | | |
| --- | --- | --- | --- | --- | --- | --- | --- | --- | --- |
| **ST** | **PorA VR1, VR2** | **FetA** | **fHbp** | | **NHBA** | **NadA** | | **N^o^ of covered strains** | **N^o^ of not covered strains** |
|  |  |  | **Variant family** | **Peptide** | **Peptide** | **Variant** | **Peptide** |  |  |
| ST-2209 (5) | 5,2 (5) | F1-5 (2) | 1 (2) | 13 (2) | 24 (2) | - (2) | - (2) | 0 | 2 |
|  |  | F1-7 (1) | 1 (1) | 13 (1) | 24 (1) | - (1) | - (1) | 1^a^ | 0 |
|  |  | F3-7 (2) | 1 (2) | 1 (1) | 24 (1) | 6 (1) | FS (1) | 1^b^ | 0 |
|  |  |  |  | 13 (1) | 24 (1) | 6 (1) | FS (1) | 1^a^ | 0 |
| ST-1383 (2) | 5,2 (2) | F1-7 (2) | 1 (2) | 13 (2) | 24 (2) | - (2) | - (2) | 2^a(1),b(1)^ | 0 |
| ST-60 (1) | 5,2 (1) | F5-5 (1) | 1 (1) | 13 (1) | 24 (1) | - (1) | - (1) | 1^a^ | 0 |
| ST-61 (1) | 5,2 (1) | F1-5 (1) | 1 (1) | 13 (1) | 24 (1) | - (1) | - (1) | 1^b^ | 0 |
| ST-8627 (1) | 19,15-1 (1) | F1-7 (1) | 1 (1) | 13 (1) | 24 (1) | - (1) | - (1) | 0 | 1 |
| ST-8833 (1) | 5,2 (1) | F5-5 (1) | 1 (1) | 13 (1) | 24 (1) | - (1) | - (1) | 1^a^ | 0 |
| ST-8834 (1) | 21,16 (1) | F1-55 (1) | 1 (1) | 13 (1) | 25 (1) | - (1) | - (1) | 0 | 1 |
| ST-8837 (1) | 5, 2-28 (1) | F5-12 (1) | 2 (1) | 24 (1) | 469 (1) | - (1) | - (1) | 0 | 1 |
| ST-8841 (1) | 5,2 (1) | F1-5 (1) | 1 (1) | 13 (1) | 24 (1) | - (1) | - (1) | 1^a^ | 0 |
| ST-8858 (1) | 5,2 (1) | F1-5 (1) | 1 (1) | 13 (1) | 24 (1) | - (1) | - (1) | 1^a^ | 0 |
| ST-8860 (1) | 5,2 (1) | F1-5 (1) | 1 (1) | 13 (1) | 24 (1) | - (1) | - (1) | 0 | 1 |
| **TOTAL** | **16** | | | | | | | **10** | **6** |

| **cc 865 (n)** | | | | | | | | | |
| --- | --- | --- | --- | --- | --- | --- | --- | --- | --- |
| **ST** | **PorA VR1, VR2** | **FetA** | **fHbp** | | **NHBA** | **NadA** | | **N^o^ of covered strains** | **N^o^ of not covered strains** |
|  |  |  | **Variant family** | **Peptide** | **Peptide** | **Variant** | **Peptide** |  |  |
| ST-4237 (5) | 7-4,1 (5) | F1-6 (5) | 2 (5) | 16 (1) | 130 (1) | - (1) | - (1) | 1^a^ | 0 |
|  |  |  |  | 25 (3) | 130 (2) | - (2) | - (2) | 2^a^ | 0 |
|  |  |  |  |  | 355 (1) | - (1) | - (1) | 1^a^ | 0 |
|  |  |  |  | 511 (1) | 130 (1) | - (1) | - (1) | 1^a^ | 0 |
| ST-3327 (1) | 21,16-36 (1) | F5-9 (1) | 1 (1) | 1 (1) | 24 (1) | - (1) | - (1) | 1^b^ | 0 |
| ST-8822 (1) | 7-4,1 (1) | F1-6 (1) | 2 (1) | 25 (1) | 130 (1) | - (1) | - (1) | 1^a^ | 0 |
| ST-8832 (1) | 7-4,1 (1) | F5-1 (1) | 1 (1) | 13 (1) | 17 (1) | - (1) | - (1) | 1^a^ | 0 |
| ST-8838 (1) | 19,15 (1) | F1-6 (1) | 2 (1) | 25 (1) | 130 (1) | - (1) | - (1) | 0 | 1 |
| ST-8922 (1) | 7-4,1 (1) | F5-1 (1) | 2 (1) | 25 (1) | 118 (1) | - (1) | - (1) | 1^a^ | 0 |
| **TOTAL** | **10** | | | | | | | **9** | **1** |

| **cc 35 (n)** | | | | | | | | | |
| --- | --- | --- | --- | --- | --- | --- | --- | --- | --- |
| **ST** | **PorA VR1, VR2** | **FetA** | **fHbp** | | **NHBA** | **NadA** | | **N^o^ of covered strains** | **N^o^ of not covered strains** |
|  |  |  | **Variant family** | **Peptide** | **Peptide** | **Variant** | **Peptide** |  |  |
| ST-35 (2) | 22-1,14 (2) | F1-55 (1) | 2 (1) | 16 (1) | 21 (1) | - (1) | - (1) | 1^a^ | 0 |
|  |  | F4-1 (1) | 2 (1) | 16 (1) | 21 (1) | - (1) | - (1) | 0 | 1 |
| ST-457 (2) | 22-1,14 (2) | F4-1 (2) | 2 (2) | 16 (2) | 21 (2) | - (2) | - (2) | 0 | 2 |
| ST-8753 (1) | 22-1,14 (1) | Not amplified (1) | 1 (1) | 252 (1) | 21 (1) | - (1) | - (1) | 1^b^ | 0 |
| ST-8867 (1) | 22-1,14 (1) | F4-1 (1) | 2 (1) | 16 (1) | 21 (1) | - (1) | - (1) | 0 | 1 |
| **TOTAL** | **6** | | | | | | | **2** | **4** |

| **cc 364 (n)** | | | | | | | | | |
| --- | --- | --- | --- | --- | --- | --- | --- | --- | --- |
| **ST** | **PorA VR1, VR2** | **FetA** | **fHbp** | | **NHBA** | **NadA** | | **N^o^ of covered strains** | **N^o^ of not covered strains** |
|  |  |  | **Variant family** | **Peptide** | **Peptide** | **Variant** | **Peptide** |  |  |
| ST-8211 (2) | 12-1,13 (1) | F1-5 (1) | 2 (1) | 34 (1) | 6 (1) | - (1) | - (1) | 0 | 1 |
|  | 12-1,13-1 (1) | F1-5 (1) | 3 (1) | 174 (1) | 6 (1) | - (1) | - (1) | 1^a^ | 0 |
| ST-8759 (1) | 18-1,34 (1) | F5-12 (1) | 1 (1) | 13 (1) | 367 (1) | - (1) | - (1) | 0 | 1 |
| ST-9265 (1) | 22,9 (1) | F5-1 (1) | 3 (1) | 31 (1) | 17 (1) | - (1) | - (1) | 0 | 1 |
| **TOTAL** | **4** | | | | | | | **1** | **3** |

| **cc 103 (n)** | | | | | | | | | |
| --- | --- | --- | --- | --- | --- | --- | --- | --- | --- |
| **ST** | **PorA VR1, VR2** | **FetA** | **fHbp** | | **NHBA** | **NadA** | | **N^o^ of covered strains** | **N^o^ of not covered strains** |
|  |  |  | **Variant family** | **Peptide** | **Peptide** | **Variant** | **Peptide** |  |  |
| ST-4963 (1) | 18-7,9 (1) | F3-9 (1) | 2 (1) | 25 (1) | 24 (1) | - (1) | - (1) | 1^a^ | 0 |
| ST-8747 (1) | 18-1,1 (1) | F1-5 (1) | 1 (1) | 373 (1) | 13 (1) | - (1) | - (1) | 0 | 1 |
| **TOTAL** | **2** | | | | | | | **1** | **1** |

| **cc 18 (n)** | | | | | | | | | |
| --- | --- | --- | --- | --- | --- | --- | --- | --- | --- |
| **ST** | **PorA VR1, VR2** | **FetA** | **fHbp** | | **NHBA** | **NadA** | | **N^o^ of covered strains** | **N^o^ of not covered strains** |
|  |  |  | **Variant family** | **Peptide** | **Peptide** | **Variant** | **Peptide** |  |  |
| ST-8757 (2) | 22,26-7 (2) | F1-5 (2) | 1 (2) | 37 (2) | 354 (2) | - (2) | - (2) | 2^a(1),b(1)^ | 0 |
| **TOTAL** | **2** | | | | | | | **2** | **0** |

| **cc 8 (n)** | | | | | | | | | |
| --- | --- | --- | --- | --- | --- | --- | --- | --- | --- |
| **ST** | **PorA VR1, VR2** | **FetA** | **fHbp** | | **NHBA** | **NadA** | | **N^o^ of covered strains** | **N^o^ of not covered strains** |
|  |  |  | **Variant family** | **Peptide** | **Peptide** | **Variant** | **Peptide** |  |  |
| ST-66 (1) | 5,2 (1) | F3-9 (1) | 2 (1) | 548 (1) | 20 (1) | 2/3 (1) | 7 (1) | 1^c^ | 0 |
| **TOTAL** | **1** | | | | | | | **1** | **0** |

| **cc 11 (n)** | | | | | | | | | |
| --- | --- | --- | --- | --- | --- | --- | --- | --- | --- |
| **ST** | **PorA VR1, VR2** | **FetA** | **fHbp** | | **NHBA** | **NadA** | | **N^o^ of covered strains** | **N^o^ of not covered strains** |
|  |  |  | **Variant family** | **Peptide** | **Peptide** | **Variant** | **Peptide** |  |  |
| ST-11 (1) | 5-1,10-8 (1) | F3-6 (1) | 1 (1) | 10 (1) | 20 (1) | 2/3 (1) | IS1301^5^ (1) | 1^e^ | 0 |
| **TOTAL** | **1** | | | | | | | **1** | **0** |

| **cc 23 (n)** | | | | | | | | | |
| --- | --- | --- | --- | --- | --- | --- | --- | --- | --- |
| **ST** | **PorA VR1, VR2** | **FetA** | **fHbp** | | **NHBA** | **NadA** | | **N^o^ of covered strains** | **N^o^ of not covered strains** |
|  |  |  | **Variant family** | **Peptide** | **Peptide** | **Variant** | **Peptide** |  |  |
| ST-23 (1) | 5-2,10-2 (1) | F1-22 (1) | 2 (1) | 104 (1) | 8 (1) | - (1) | - (1) | 0 | 1 |
| **TOTAL** | **1** | | | | | | | **0** | **1** |

| **NA^2^** | | | | | | | | | |
| --- | --- | --- | --- | --- | --- | --- | --- | --- | --- |
| **ST** | **PorA VR1, VR2** | **FetA** | **fHbp** | | **NHBA** | **NadA** | | **N^o^ of covered strains** | **N^o^ of not covered strains** |
|  |  |  | **Variant family** | **Peptide** | **Peptide** | **Variant** | **Peptide** |  |  |
| ST-1434 (2) | 7-2,13-1 (2) | F1-62 (1) | 2 (1) | 16 (1) | 306 (1) | - (1) | - (1) | 1^a^ | 0 |
|  |  | F5-2 (1) | 1 (1) | 215 (1) | 306 (1) | - (1) | - (1) | 1^b^ | 0 |
| ST-3823 (2) | 22,9 (1) | F5-8 (1) | 2 (1) | 16 (1) | 306 (1) | - (1) | - (1) | 0 | 1 |
|  | 19,15-1 (1) | F5-5 (1) | 2 (1) | 16 (1) | 306 (1) | - (1) | - (1) | 0 | 1 |
| ST-3934 (2) | 22,9 (2) | F5-12 (2) | 2 (2) | 19 (2) | 17 (2) | - (2) | - (2) | 2^a^ | 0 |
| ST-2003 (1) | 17,9 (1) | F1-7 (1) | 2 (1) | 19 (1) | 366 (1) | - (1) | - (1) | 0 | 1 |
| ST-4953 (1) | 7-12,14 (1) | F1-7 (1) | 2 (1) | 16 (1) | 20 (1) | - (1) | - (1) | 1^a^ | 0 |
| ST-4954 (1) | 5,2 (1) | F5-2 (1) | 1 (1) | 14 (1) | 30 (1) | - (1) | - (1) | 1^b^ | 0 |
| ST-5562 (1) | 22,9 (1) | F5-1 (1) | 2 (1) | 19 (1) | 17 (1) | - (1) | - (1) | 1^a^ | 0 |
| ST-7006 (1) | 7-2,14 (1) | F1-7 (1) | 2 (1) | 16 (1) | 307 (1) | - (1) | - (1) | 1^a^ | 0 |
| ST-7008 (1) | 22,9 (1) | F1-55 (1) | 2 (1) | 19 (1) | 17 (1) | - (1) | - (1) | 1^a^ | 0 |
| ST-8567 (1) | 7-1,1 (1) | F3-6 (1) | 1 (1) | 35 (1) | 6 (1) | - (1) | - (1) | 0 | 1 |
| ST-8626 (1) | 19,15-1 (1) | F1-55 (1) | 1 (1) | 108 (1) | 17 (1) | 4/5 (1) | 21 (1) | 1^e^ | 0 |
| ST-8628 (1) | 17,9 (1) | F1-7 (1) | 2 (1) | 551 (1) | 366 (1) | - (1) | - (1) | 0 | 1 |
| ST-8749 (1) | 22,9 (1) | F1-55 (1) | 3 (1) | 47 (1) | 17 (1) | - (1) | - (1) | 1^a^ | 0 |
| ST-8752 (1) | 22,9 (1) | F1-55 (1) | 1 (1) | 1 (1) | 17 (1) | - (1) | - (1) | 1^b^ | 0 |
| ST-8754 (1) | 7-2,14 (1) | F5-2 (1) | 1 (1) | 14 (1) | 30 (1) | 4/5 (1) | 21 (1) | 1^b^ | 0 |
| ST-8755 (1) | 7-12,14 (1) | F5-8 (1) | 1 (1) | 13 (1) | 20 (1) | - (1) | - (1) | 1^a^ | 0 |
| ST-8760 (1) | 7-36,14 (1) | F1-21 (1) | 1 (1) | 13 (1) | 20 (1) | - (1) | - (1) | 1^e^ | 0 |
| ST-8821 (1) | 7-4,1 (1) | F1-6 (1) | 2 (1) | 25 (1) | 130 (1) | - (1) | - (1) | 0 | 1 |
| ST-8823 (1) | 19,15-1 (1) | F5-2 (1) | 3 (1) | 532 (1) | 30 (1) | 4/5 (1) | 21 (1) | 0 | 1 |
| ST-8830 (1) | 22,9 (1) | F5-7 (1) | 2 (1) | 19 (1) | 17 (1) | 4/5 (1) | FS (1) | 1^a^ | 0 |
| ST-8836 (1) | 7-12,14 (1) | F3-9 (1) | 1 (1) | 13 (1) | 20 (1) | - (1) | - (1) | 1^a^ | 0 |
| ST-8842 (1) | 19,15-1 (1) | F5-2 (1) | 1 (1) | 14 (1) | 30 (1) | 4/5 (1) | 21 (1) | 0 | 1 |
| ST-8843 (1) | 19,15-1 (1) | F5-2 (1) | 1 (1) | 1 (1) | 470 (1) | 4/5 (1) | 21 (1) | 1^e^ | 0 |
| ST-8844 (1) | 22,9 (1) | F5-12 (1) | 1 (1) | 1 (1) | 17 (1) | - (1) | - (1) | 1^e^ | 0 |
| ST-8845 (1) | 7-12,14 (1) | F1-7 (1) | 1 (1) | 13 (1) | 20 (1) | - (1) | - (1) | 1^a^ | 0 |
| ST-8846 (1) | 19,15-1 (1) | F5-2 (1) | 1 (1) | 14 (1) | 30 (1) | - (1) | - (1) | 0 | 1 |
| ST-8847 (1) | Deleted,16-16 (1) | F5-2 (1) | 3 (1) | 31 (1) | 370 (1) | - (1) | - (1) | 1^a^ | 0 |
| ST-8865 (1) | 22,9 (1) | F1-55 (1) | 3 (1) | 45 (1) | 24 (1) | - (1) | - (1) | 1^a^ | 0 |
| ST-8866 (1) | 18,25-7 (1) | F3-7 (1) | 2 (1) | 24 (1) | 2 (1) | - (1) | - (1) | 1^a^ | 0 |
| ST-8870 (1) | 7,30 (1) | F4-1 (1) | 2 (1) | 19 (1) | 17 (1) | - (1) | - (1) | 0 | 1 |
| ST-8871 (1) | 21,16 (1) | F4-1 (1) | 1 (1) | 13 (1) | 468 (1) | - (1) | - (1) | 0 | 1 |
| ST-8874 (1) | 22,9 (1) | F3-7 (1) | 2 (1) | 19 (1) | 17 (1) | - (1) | - (1) | 1^a^ | 0 |
| ST-8890 (1) | 7-4,1 (1) | F1-6 (1) | 2 (1) | 25 (1) | 130 (1) | - (1) | - (1) | 1^a^ | 0 |
| ST-8939 (1) | 7-2,4-9 (1) | F1-7 (1) | 1 (1) | 13 (1) | 20 (1) | - (1) | - (1) | 1^a^ | 0 |
| ST-8953 (1) | 19,15-1 (1) | F1-5 (1) | 1 (1) | 108 (1) | 30 (1) | - (1) | - (1) | 0 | 1 |
| ST-9191 (1) | 19,15 (1) | F4-1 (1) | 1 (1) | 213 (1) | 465 (1) | - (1) | - (1) | 1^a^ | 0 |
| ST-960 (1) | 12-1,13-9 (1) | F1-52 (1) | 2 (1) | 18 (1) | 304 (1) | - (1) | - (1) | 0 | 1 |
| **TOTAL** | **40** | | | | | | | **27** | **13** |

^1^ Strains were defined as covered by 4CMenB/Bexsero® vaccine if they presented PorA P1.4 or had a RP greater than the PBT for fHbp, NHBA and/or NadA: ^a^ strains covered by NHBA (NHBA MATS RP ≥ 0.294), ^b^ strains covered by fHbp (fHbp MATS RP > 0.021), ^c^ strains covered by NadA (NadA MATS RP > 0.009), ^d^ strains covered by PorA (presence of PorA VR2=4), ^e^ strains covered by NHBA and fHbp, ^f^ strains covered by NHBA and PorA, ^g^ strains covered by fHbp and PorA, ^h^ strains covered by fHbp and NadA, ^i^ strains covered by NHBA, fHbp and PorA.

^2^ NA: Non assigned clonal complexes.

^3^ NadA variant/peptide -: gene not present.

^4^ FS: gene with a frameshift mutation resulting in a premature stop codon.

^5^ IS: gene disrupted by an insertion sequence (IS1301).

The specific antigens included in the 4CMenB/Bexsero® vaccine are labeled in red.
